# Noninvasive imaging of radiolabeled exosome-mimetic nanovesicle using ^99m^Tc-HMPAO

**DOI:** 10.1038/srep15636

**Published:** 2015-10-26

**Authors:** Do Won Hwang, Hongyoon Choi, Su Chul Jang, Min Young Yoo, Ji Yong Park, Na Eun Choi, Hyun Jeong Oh, Seunggyun Ha, Yun-Sang Lee, Jae Min Jeong, Yong Song Gho, Dong Soo Lee

**Affiliations:** 1 Department of Nuclear Medicine, Seoul National University College of Medicine, Seoul, Korea; 2 Department of Molecular Medicine and Biopharmaceutical Sciences, Graduate School of Convergence Science and Technology, and College of Medicine or College of Pharmacy, Seoul National University, Seoul, Korea; 3 Department of Life Sciences, Pohang University of Science and Technology, Pohang, Gyeongbuk 790-784, Korea; 4 Krefting Research Centre, Department of Internal Medicine and Clinical Nutrition, University of Gothenburg, 405 30 Gothenburg, Sweden

## Abstract

Exosomes known as nano-sized extracellular vesicles attracted recent interests due to their potential usefulness in drug delivery. Amid remarkable advances in biomedical applications of exosomes, it is crucial to understand *in vivo* distribution and behavior of exosomes. Here, we developed a simple method for radiolabeling of macrophage-derived exosome-mimetic nanovesicles (ENVs) with ^99m^Tc-HMPAO under physiologic conditions and monitored *in vivo* distribution of ^99m^Tc-HMPAO-ENVs using SPECT/CT in living mice. ENVs were produced from the mouse RAW264.7 macrophage cell line and labeled with ^99m^Tc-HMPAO for 1 hr incubation, followed by removal of free ^99m^Tc-HMPAO. SPECT/CT images were serially acquired after intravenous injection to BALB/c mouse. When ENVs were labeled with ^99m^Tc-HMPAO, the radiochemical purity of ^99m^Tc-HMPAO-ENVs was higher than 90% and the expression of exosome specific protein (CD63) did not change in ^99m^Tc-HMPAO-ENVs. ^99m^Tc-HMPAO-ENVs showed high serum stability (90%) which was similar to that in phosphate buffered saline until 5 hr. SPECT/CT images of the mice injected with ^99m^Tc-HMPAO-ENVs exhibited higher uptake in liver and no uptake in brain, whereas mice injected with ^99m^Tc-HMPAO showed high brain uptake until 5 hr. Our noninvasive imaging of radiolabeled-ENVs promises better understanding of the *in vivo* behavior of exosomes for upcoming biomedical application.

Exosomes are nano-sized extracellular vesicles which are produced by most of the cell types. Exosomes carry a subset of genetic materials and proteins to play key roles in delivery of the biological information between cells^1^,^2^. Since exosomes were found initially to play a role in disposing unnecessary proteins such as transferrin receptor during reticulocyte maturation 30 years ago, a variety of functional roles have been elucidated^3^,^4^. Especially, recently-alleged biological roles of exosomes ranged from their involvement in normal physiologic functions such as stem cell maintenance, immune modulation and tissue homeostasis to their contribution to the pathophysiology of several diseases^5^. In addition, exosomes also delivered variable sets of bioactive materials specific for parent cell-types and mediated cellular activities of recipient cells^6^.

Exosomes can be used as drug carriers by applying their role of *in vivo* intercellular communication. As natural endogenous nanoparticles, the autologous exosomes have the advantages of immune-tolerance and stability in circulation systems^7^. In addition, exosomes derived from different cells have target-specific homing effect even to achieve active targeting^8^,^9^. To enhance their targeting potency, *ex vivo* cell-derived exosomes were engineered to carry specific surface proteins^10^. According to these advantages, *ex vivo* cell-derived exosomes were tried to be used as nanocarriers. For example, exosomes were used to target cancer tissue by transporting therapeutic nucleic acids in animal experiments^11^. Even in the clinical setting, cancer antigen-pulsed autologous exosomes isolated from patients were already applied for cancer immunotherapy^12^,^13^. However, one major drawback of exosome-based nanocarriers was the small amount of natural exosomes. This low yield limits *in vivo* application where large amount of exosomes are necessary.

Recently exosome-mimetic nanovesicles (ENVs) were collected from whole cells as alternatives to the natural exosomes also collected from the cells^14^. This exosome-mimetic technology overcame several limitations of natural exosomes for the application of practical drug delivery, which included low yield (0.3 to 0.5 μg of exosomes per 10^6^ dendritic cells in 24 h)^15^ and difficulties in purification process^16^. These ENVs have similar characteristics with natural exosomes in terms of size, morphology and protein contents. ENVs were easily produced with 100-fold higher amount than naturally collected exosomes, which enabled therapeutic application^14^. In addition, ENVs shared the similar homing properties to the natural exosomes, which was revealed by showing accumulation of macrophage-derived ENVs in the tumors visualized by *in vivo* fluorescence imaging^14^.

Considering that there is a variety of cell-type specific exosomes, *in vivo* behaviors of these exosomes may be highly varied. However, there were very few effective methods reported to investigate the *in vivo* behavior of these exosomes, which also became an obstacle for the biomedical application of these exosomes or ENVs. A breakthrough to investigate *in vivo* distribution and trace the exosomes is highly desired. To date, fluorescence imaging was tried to trace the whereabouts of the exosomes and to investigate the distribution of cell-derived membrane vesicles^17^,^18^,^19^,^20^. Although *in vivo* optical imaging is a simple tool for *in vitro* and *in vivo* study, it has intrinsic limitations including difficulties in quantification and poor tissue penetration depth. Radioisotope (RI) labeling and nuclear imaging can easily overcome these limitations and can be applied to trace exosomes *in vivo*^21^,^22^.

In this study, we developed a simple radiolabeling method for ENVs with ^99m^Tc- hexamethylpropyleneamineoxime (HMPAO) under physiologic condition, and visualized *in vivo* distribution of ^99m^Tc-HMPAO-ENVs using SPECT/CT. A scheme for radiolabeling of ENVs using ^99m^Tc-HMPAO was summarized in Fig. 1. ^99m^Tc-HMPAO is known as uncharged and highly lipophilic radiotracer, which has been widely used for cell labelling. It is well known that intracellular glutathione converts ^99m^Tc-HMPAO to the hydrophilic form to be trapped inside cells. Based on this property, ^99m^Tc-HMPAO has been clinically used for white blood cell (WBC) labeling for inflammation imaging^23^. Since ^99m^Tc-HMPAO is an easily accessible radiotracer in the clinical setting, our approach is expected to facilitate biomedical application of exosomes by easily disclosing the whereabouts of exosomes or ENVs.

## Results

### Radiolabeling of ENVs with ^99m^Tc-HMPAO

ENVs produced from mouse Raw 264.7 macrophage cell line were purified from interface layer through OptiPrep density gradient fractionation. When we measured glutathione in ENVs for ^99m^Tc-HMPAO labeling, we found 3.15 ± 0.16 nmol of glutathione in 0.1 mg of ENVs (Supplementary Fig. 1).

**Figure 2:**
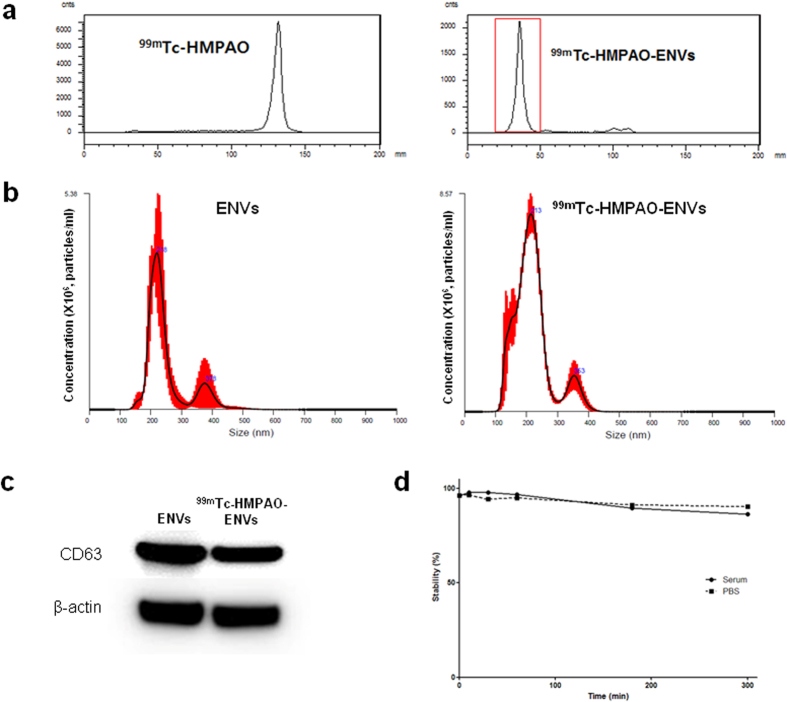
Radiochemical purification of ^99m^Tc-HMPAO-ENVs and characteristics . (**a**) ^99m^Tc-HMPAO and ^99m^Tc-HMPAO-ENVs were analyzed by instant thin layered chromatography (ITLC) using Whatman No. 1 paper. The radiochemical purify was analyzed right after removing ^99m^Tc-HMPAO. The radiochemical purify was more than 95%. (**b**) Average size distribution of non-labeled ENVs and ^99m^Tc-HMPAO-ENVs was examined by nanoparticle tracking analysis (NTA). There was no significant difference for the size distribution between non-labeled ENVs and ^99m^Tc-HMPAO-labeled ENVs. (**c**) An exosomal marker, CD63, was measured to check ENVs before and after ^99m^Tc-HMPAO radiolabeling by Western blot. Amount of exosomal protein in its expression was not changed, showing high expression of CD63 which was similar with protein expression in unlabeled ENVs. (**d**) Stability of ^99m^Tc-HMPAO-ENVs was examined in human serum and PBS. The level of stability was analyzed by ITLC.

ENVs were incubated with ^99m^Tc-HMPAO for labeling under mild condition (in pH 7, PBS buffer) in order to prevent the labeled-ENVs from unexpected aggregation. After labeling, purification was performed in two methods to remove free ^99m^Tc-HMPAO, as described in Methods. Radiochemical purity was 99.6 ± 3.3% after the size exclusion column was applied (Fig. 2a), and 93.7 ± 5.4% after purification with exosome exclusive spin column with centrifugation. The procedure time of exosome-spine column method was 3 min, which was 10 times less than the size exclusion column method (Table 1). Considering both methods showed comparable radiochemical purity while exosome exclusive spin column method took less time, the exosome-spin column method was used for further characterization study, SPECT/CT imaging and *ex vivo* biodistribution study.

### Characterization of ENVs after ^99m^Tc-HMPAO radiolabeling

Size distribution of unlabeled ENVs and ^99m^Tc-HMPAO-ENVs was measured using nanoparticle tracking analysis (NTA). The average diameter of unlabeled ENVs was 218 ± 8.2 nm, and that of ^99m^Tc-HMPAO-ENVs was 213 ± 6.8 nm (Fig. 2b), showing no significant changes after ^99m^Tc-HMPAO radiolabeling. In order to confirm whether the exosome-specific protein contents of ENVs changed after ^99m^Tc-HMPAO radiolabeling, an exosomal marker, CD63, was measured by western blot analysis. Amount of protein in ^99m^Tc-HMPAO-ENVs did not change, showing high expression of CD63 similar to that in unlabeled ENVs (Fig. 2c). We performed the serum stability test for ^99m^Tc-HMPAO-ENVs. ^99m^Tc-HMPAO-ENVs showed comparable serum stability with that in PBS, maintaining around 90% until 5 hr (Fig. 2d).

### *In vivo* SPECT/CT images and biodistribution of ^99m^Tc-HMPAO-ENVs

**Figure 3:**
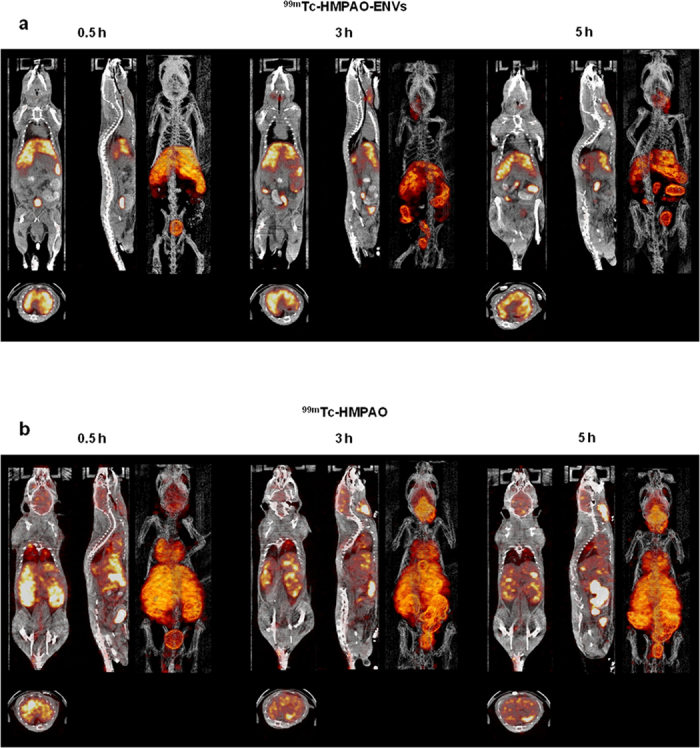
*In vivo* SPECT/CT images of ^99m^Tc-HMPAO-ENVs injected in mice. After intravenous injection of ^99m^Tc-HMPAO-ENVs or ^99m^Tc-HMPAO, SPECT/CT images were acquired at 30 min, 3 hr, and 5 hr in BALB/c mice. (**a)** The SPECT/CT imaging showed the significantly intense uptake of ^99m^Tc-HMPAO-ENVs in the liver and radioactivity in the salivary glands and intestine until 5 hr. (**b**) In contrast, high brain uptake and the delayed salivary glands uptake were observed in ^99m^Tc-HMPAO-injected mouse group.

*In vivo* imaging of ^99m^Tc-HMPAO-ENVs and ^99m^Tc-HMPAO were acquired by SPECT/CT. Within 30 min after injection, ^99m^Tc-HMPAO-ENVs were taken up mainly by the liver and spleen (Fig. 3a). Of note, no significant brain uptake was observed, compared with the significant brain accumulation in the mice with ^99m^Tc-HMPAO-only administration at 30 min images (Fig. 3b). At 3 hr after the injection, ^99m^Tc-HMPAO-ENVs accumulated in the salivary glands. In contrast, in the mice with ^99m^Tc-HMPAO-only administration, radioactivity was relatively low in the liver and spleen, and this radioactivity decreased gradually until 5 hr (Fig. 3b). The intense uptake in the brain and intestine was observed immediately after the injection and the activity was remained until 5 hr. Salivary gland uptake was high in ^99m^Tc-HMPAO-only administered group. Quantitative volume-of-interest analysis based on SPECT/CT images revealed similar distribution pattern to visual findings (Supplementary Fig. 2).

We additionally acquired SPECT/CT imaging of ^99m^Tc-HMPAO-ENVs obtained from HB1.F3 human neural stem cells to test whether this method could be used in different type of cells. Just like the ENVs obtained from Raw 264.7 macrophage cells, the ENVs from neural stem cells showed the similar stability in the serum, and after systemic administration, they accumulated in the liver and spleen within 30 min and the uptakes in salivary glands increased gradually (Supplementary Fig. 3a,b). We also performed production of natural exosomes using Raw 264.7 macrophage cells and labeled them with the same method. After 3 weeks of culture and preparation, we could produce half the amount of exosomes than the ENVs, and SPECT/CT disclosed the distribution of ^99m^Tc-HMPAO labeled natural exosomes. Low sensitivity due to lower amount of exosomes and radioactive label than ENVs made the images poor, but accumulation pattern was similar to that of ENVs at 3 hr. ^99m^Tc-HMPAO labeled natural exosomes accumulated in liver and spleen and then bowel (Supplementary Fig. 3c).

**Figure 4:**
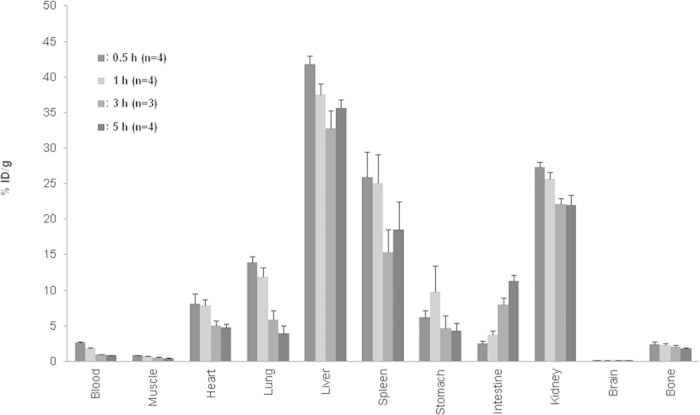
Biodistribution of ^99m^Tc-HMPAO-ENVs. Mice were sacrificed at 0.5, 1, 3, 5 hr after ^99m^Tc-HMPAO ENVs injection and radioactivity was measured for quantitative analysis of biodistribution (n = 4 for groups sacrificed at 0.5, 1 and 5 hr; n = 3 for a group sacrificed at 3 hr). As shown in SPECT images, ^99m^Tc-HMPAO-ENVs were highly accumulated in liver, spleen and kidneys. Note that no significant uptake in brain unlike ^99m^Tc-HMPAO used as a brain perfusion tracer. %ID/g represents counts of radioactivity normalized by injected dose and weights of organ. Data are presented as mean ± S.D.

Mice were sacrificed after 0.5, 1, 3, and 5 h of ^99m^Tc-HMPAO-ENVs to investigate *in vivo* distribution using *ex vivo* quantitation of radioactivity. The distribution pattern was different particularly in the liver and brain between ^99m^Tc-HMPAO-ENVs and ^99m^Tc-HMPAO-only administered groups (Fig. 4).

## Discussion

Exosome-based drug delivery receives great attention owing to merits as a naturally obtained nanoparticle which is able to bypass many issues of nanoparticles including immune activation, off-target effect and *in vivo* degradation^6^. Although cell-derived exosomes have showed therapeutic effects on specific inflammatory or cancer condition^14^,^24^, it remains unknown whether the injected exosomes are effectively delivered to the target tissue. To achieve breakthrough in exosome-based therapy, *in vivo* imaging is necessary.

In this study, we carried out radiolabeled-exosome imaging using ENVs which share biological features with naturally derived exosomes^14^. To investigate whereabouts and kinetics of administered ENVs or exosomes, imaging should be performed in living animals which can provide quantitative information even of the deep organs. Since exosomes are natural nanoparticles, distinct from artificially-made nanoparticles, labeling should be performed under biologically-inert and conservative condition. Until now, in previous studies fluorescence reporters or highly lipophilic dyes were used to track the *in vitro* and *in vivo* behavior in cells or living animals^17^,^25^,^26^. These methods had intrinsic limitations in light penetration depth and quantitation. MRI can overcome the disadvantage of optical imaging and modified liposomes were proposed as artificial exosomes which were constructed to contain surface markers and super paramagnetic labels for magnetic resonance imaging^27^, however, there was no suggestion how to make exosomes or ENVs with paramagnetic materials. Radionuclide labeling and imaging could be an alternative because of their high sensitivity, noninvasiveness and quantification of biodistribution. Moreover, *ex vivo* biodistribution study using radioactivity as we employed in this study accurately provides the absolute information of whereabouts after sacrifice of the animals even in the small organs with high sensitivity. To our knowledge, two studies have tried to develop radioisotope-labeled exosome to evaluate biodistribution, however, they only showed *ex vivo* biodistribution and a study engaged streptavidin reporter which needed genetically modified cells^21^,^22^. To achieve *in vivo* radionuclide-labeled exosome imaging under physiologic condition, we employed ^99m^Tc-HMPAO. By reaction of ^99m^Tc-HMPAO with sulfhydryl groups of the glutathione abundantly existing in cells, ^99m^Tc-HMPAO became hydrophilic compounds and trapped within cells. Because of this property, ENVs composed of lipid bilayer were expected to be simply labeled with ^99m^Tc-HMPAO under physiologic condition. This approach was introduced in glutathione-contained liposome labeling and was used for liposome imaging^28^,^29^. As liposomes and exosomes consist of lipid bilayer, and moreover, exosomes had glutathione inside the exosomes or ENVs, the ^99m^Tc-HMPAO labeling resulted in comparable excellent radiochemical purity (higher than 95%). Furthermore, approximately 90% of entrapped ^99m^Tc in ENVs persisted within the exosomes and ENVs in the serum until 5 hr. When erythrocytes and platelets were labeled with ^99m^Tc-HMPAO, spontaneous release rate for 1 h ranged from 5% to 10%^30^,^31^, which corresponded to that of ENVs.

^99m^Tc-HMPAO labeling for ENVs could be performed under physiologic condition not to change their properties. In the *in vitro* study, CD63, an exosome marker, did not change after labeling. We also found that average size distribution did not change after labeling of ENVs with ^99m^Tc-HMPAO. A small fraction of ENVs which were smaller than average size was identified after labeling (Fig. 2b), which could mean that a small portion of ENVs would have been damaged during labeling procedures. Fortunately, the overall size distribution was the same after the procedures. In the previous our work (unpublished), ENVs were modified with SCN-Bn-NOTA, which is a bifunctional chelating agent, and then labeled with ^68^Ga. After the ^68^Ga labeling, the size of ENVs increased up to 530 nm probably due to aggregations which resulted in the accumulation in lung (Supplementary Fig. 4). We speculated that labeling condition was closely related to aggregations and labeling for ENVs absolutely need the physiologic condition. In this study, the size of ^99m^Tc-HMPAO-labeled ENVs did not dramatically change as in ^68^Ga labeling after labeling. *In vivo* SPECT/CT images of ^99m^Tc-HMPAO-ENVs proved no lung accumulation suggesting negligible aggregations during labeling procedures and *in vivo* after systemic injection.

In this study, we adopted two different purification methods based on either size exclusion column or exosome exclusive spin column. Both methods showed comparable labeling efficiency with rapid radiolabeling time, but in the case of size exclusion column method, large amount of ENVs was lost during elution. In addition, the size exclusion column method takes 10 times longer until final elution than spin column-based centrifugation (30 min vs. 3 min, Table 1). Although the loading volume of exosome in spin column is limited (<100 μl), we suggest that spin column-based centrifugation would be a better method of radiolabeled ENVs isolation in terms of rapid and high yield of ^99m^Tc-HMPAO-ENVs.

We found that ^99m^Tc-labeled-ENVs could provide sensitive, quantitative and reproducible imaging. ^99m^Tc-HMPAO-ENVs administrated mouse showed high accumulation of injected ENVs in liver and spleen at 30 min, which was about 2–3 folds higher than ^99m^Tc-HMPAO-only administered mouse. The uptake of ^99m^Tc-HMPAO-ENVs in the liver and spleen could be a result of phagocytosis in mononuclear phagocyte systems. Since ENVs in our experiments were derived from mouse macrophage cells, they could be actively captured by host-derived cells abundant in liver and spleen^32^. SPECT/CT images of ^99m^Tc-HMPAO-ENVs obtained from human neural stem cells revealed similar accumulation pattern to those obtained from Raw 264.7 cells. Besides, similar-sized liposomes were reported to accumulate passively in mononuclear phagocyte systems^33^, and thus, the accumulation mechanism of ENVs might be soon understood. Distribution study of natural exosomes was also feasible though the yield of exosomes was not sufficient for radiolabeling and purification. To obtain SPECT/CT images of natural exosomes, large amount of cells were needed to obtain minimum amount of natural exosomes. Despite this difficulty, we found that radiolabeled natural exosomes also showed similar distribution to radiolabeled ENVs derived from macrophage and neural stem cells.

^99m^Tc-HMPAO-ENVs imaging will be useful for *in vivo* drug-delivery monitoring. In biodistribution study, brain uptake was not observed in ^99m^Tc-HMPAO-ENVs administration group, whereas the ^99m^Tc-HMPAO-only injected group showed high accumulation in the brain corresponding to previous study regarding *in vivo* kinetics of ^99m^Tc-HMPAO^34^. Radioactivity of salivary glands and bowel walls appeared at 3 hr and 5 hr in ^99m^Tc-HMPAO-ENVs administration group like ^99m^Tc-HMPAO-only injected group, which might correspond to *in vitro* stability of ^99m^Tc-HMPAO-ENVs, a result of eluted ^99m^Tc-compounds from ENVs. Moreover, the release of ^99m^Tc-compounds might be facilitated *in vivo* due to phagocytosis in mononuclear phagocyte systems. These biodistribution patterns suggested ^99m^Tc-HMPAO-ENVs can be used for tracing, particularly in early phase, considering that no brain and minimal salivary gland uptake was detected. In the future, because of eluted ^99m^Tc-compounds, accumulation of ENVs in target tissue should be carefully interpreted as in ^99m^Tc-HMPAO-labeled leukocyte imaging in the routine clinical setting^35^.

As exosomes are derived from biological tissues, they potentially have advantages in clinical use for diagnostic imaging as well as drug carriers. Many researchers have tried to exploit exosomes as potential drug delivery nanocarriers to transport chemical drug or siRNA to the disease site^7^,^36^. In spite of the limitations in delayed spontaneous release of ^99m^Tc-compounds, our approach has a wide range of applications involving brain targeting, cancer and inflammation imaging. Radiolabeled-exosomes can be used to track *in vivo* behavior of exosomes to find out where the administrated exosomes is localized and how long exosomes is retained in the target tissue. Therefore, it can contribute to understanding pharmacokinetics of the drug-loaded exosomes. Furthermore, *in vivo* imaging of exosomes derived from several types of tissues under various environmental conditions in living subjects, enables to understand in-depth biological properties and roles of exosomes. We expect that the radiolabeled-exosomes will be gaining ground as a crucial imaging technique to apply exosomes to theranostics.

We reported a simple and rapid radiolabeling method of ENVs for *in vivo* radionuclide imaging, and imaged biodistribution of ^99m^Tc-HMPAO-ENVs via SPECT/CT in living animals. Highly lipophilic ^99m^Tc-HMPAO which has been widely used for brain perfusion imaging agent was used for exosome labeling with simple and rapid reaction. Though this was a feasibility study of SPECT/CT images for exosomes, this approach has some limitations. Despite the high sensitivity of radiolabeled ENVs for noninvasive imaging (7.4–14.8 MBq of 29–64 μg radiolabeled exosomes for imaging as described in Methods), labeling procedures can be improved more to reduce the loss of ENVs during labeling and separation especially for low-yield natural exosomes although we showed the possibility of SPECT/CT imaging of natural exosomes (Supplementary Fig. 3c). Furthermore, inhomogeneous vesicles derived from cell organelles would have contaminated the ENVs as ENVs were produced by extrusion of cells via polycarbonate membrane filters. Thus, further optimization of isolation techniques is warranted. Nevertheless, *in vivo* distribution of ^99m^Tc-HMPAO-ENVs was clearly observed by SPECT/CT, showing high radioactivity in the liver and spleen. The noninvasive imaging of radiolabeled ENVs and exosomes is promising approach to investigate *in vivo* behavior of exosomes and develop exosome-based drug delivery system.

## Methods

### Cell culture

Murine macrophage Raw 264.7 cell lines isolated from peritoneal fluid of BALB/c mouse was grown in Dulbecco’s Modified Eagle Medium (DMEM, Invitrogen, Grand Island, NY). HB1.F3 human neural stem cells immortalized by retroviral transduction with the v-myc oncogene were grown in DMEM. All media were supplemented with 10% fetal bovine serum (FBS) and 10 U/mL penicillin and 10 μg/mL streptomycin (Invitrogen). All cells were cultured at 37 °C in a humidified atmosphere of 5% CO_2_. Cell culture reagents were purchased from Gibco-BRL.

### Preparation of ENVs from cell lines

After adherent Raw 264.7 and HB1.F3 cells were detached through gentle scraping, cells were resuspended at a concentration of 5×10^6^ cells/mL using phosphate-buffered saline (PBS). The cell suspension was extruded three times through 10, 5, and 1 μm-sized polycarbonate membrane filters sequentially using amini-extruder (Avanti Polar Lipids). To make density gradient step, 50% iodixanol (Axis-Shield PoC AS, Oslo, Norway) was firstly placed at the bottom of an ultracentrifuge tube with gentle loading, and 10% iodixanol was overlaid in the second step. The extruded samples were finally loaded, and then the prepared samples were spun at 100,000 × g for 2 h at 4 °C using a Beckman Coulter type 45Ti rotor (Beckman Instruments, CA). ENVs were collected from the interface of the 50% and 10% iodixanol layers. And the produced ENVs were stored at −20 °C.

### Radiolabeling of ENV with ^99m^Tc-HMPAO radiotracer

A commercially available kit of HMPAO was labeled with ^99m^TcO4^−^ without methylene blue. A vial containing 0.5 mg HMPAO and 4.0 pg SnCl_2_ was reconstituted with 1110 MBq of ^99m^TcO_4_ in 5 mL of 0.9% NaCl solution and shaken for 10 s. After 5 min of incubation, ^99m^Tc-HMPAO was immediately added to the prepared ENVs. The ENVs (100 μg/100 μl) were incubated for 1 hr with prepared 185–370 MBq of ^99m^Tc-HMPAO at room temperature. During the incubation, the tube was slowly shaken until the end of labeling.

### Isolation of ^99m^Tc-HMPAO-ENVs from free ^99m^Tc-HMPAO

Two different methods were used to remove ^99m^Tc-HMPAO from free ^99m^Tc-HMPAO-ENVs. A size exclusion column or centrifugation with exosome exclusive spin column was used for purification of radiolabeled ENVs. For the size exclusion column, ^99m^Tc-HMPAO-ENVs were isolated using PD-10 column (GE Healthcare, Milwaukee, WI, USA) eluted with 0.9% w/v NaCl solution. The eluent was collected in test tubes of each 0.5 mL fraction. Radiochemical purity was measured by thin-layer chromatography (TLC) using Whatman no.1 paper and 0.9% w/v NaCl solution as an eluent for each column. Second, an exosome exclusive spin column with centrifugation was used (MW3000, Invitrogen). After the column was hydrated using 650 μl of 0.9% w/v NaCl solution, excess interstitial fluid was removed by spinning the column at 750 × g for 2 min. The mixture of ENVs with ^99m^Tc-HMPAO was centrifuged at 750 × g for 2 min at room temperature to isolate ^99m^Tc-HMPAO-ENVs. The radioactivity was examined using an AR-2000 TLC imaging scanner (Bioscan, Washington, DC).

### Serum stability study for ^99m^Tc-HMPAO-ENVs

ENVs (100 μg, 1 mg/ml in PBS) were incubated with 185 MBq ^99m^Tc-HMPAO for 2 hr at room temperature. ^99m^Tc-HMPAO-ENVs were immersed in PBS for 1.5 h to remove initial diffusion of lipophilic ^99m^Tc-HMPAO from labeled-ENVs. ^99m^Tc-HMPAO-ENVs were purified using exosome exclusive spin column. Samples loaded into 0.22 μm-filtered human serum were incubated for 10 min, 30 min, 1 hr, 3 hr, and 5 hr. The radiochemical purity at serum condition in TLC was analyzed using 0.9% w/v NaCl solution as an eluent. Radiochemical purity was expressed as percentage of counter of ^99m^Tc-HMPAO-ENVs.

### Nanoparticle tracking analysis

The size distribution of ENVs prepared by Raw 264.7 cells was measured by the nano tracking analysis (NTA) method using a NANOSIGHT NS500 (Malvern, Grovewood road, UK) by using minor modification of manufacturer’s instruction. Samples were diluted sufficiently for the contrast and minimal background level. The quick measurement mode was performed to find the optimal condition. Then, total 5 numbers of particle motion video were recorded automatically using standard measurement mode (temperature: 22.9 °C and viscosity: 0.93 cP). The captured videos (5 videos per sample) were processed and analyzed (camera level: 4, capture duration: 60 s and detection threshold: 7). All other conditions were constant. The graphical figure was automatically drawn by software and given excel data was used for the mean value and the standard deviation.

### Glutathione assay of ENVs

Total glutathione content of ENV prepared by Raw 264.7 cells was determined by glutathione assay kit (Cayman Chemical, Ann Arbor, NY) according to the instructions of the manufacturer. Briefly, 100 μg ENVs were resuspended and lysed in 0.1% Triton X-100 for 30 min. After 15 min centrifugation at 10,000 × g at 4 °C, the supernatants were reacted with DTNB (5,5′-dithiobis-2-nitrobenzoic acid) to produce yellow-colored TNB (-thio-2-nitrobenzoic acid) and the absorbance was read at 412 nm. To determine the concentration of glutathione, the reactions were carried out with standard samples with known glutathione concentration. Three independent tests were carried out.

### Western blot analysis

The concentration of prepared ENVs or ^99m^Tc-HMPAO-ENVs was quantified using BCA protein quantification kit (Thermo Scientific, MA). ENVs or ^99m^Tc-HMPAO-ENVs with equal amounts of protein were transferred onto PVDF membranes (Millipore, MA). PVDF membranes were blocked for 2 h at room temperature in Tris-buffered saline (TBS) containing 0.1% Tween20 and 5% nonfat dried milk. PVDF membrane was probed with primary antibodies against anti-rabbit CD63 (1:1000 dilution; Santa Cruz Biotechnology, TX) at 4 °C for overnight. PVDF Membranes were incubated with HRP-labeled anti-rabbit secondary antibody for 2 hr at RT, and immunochemical detection was performed using chemiluminescence ECL detection system. Three independent experiments were performed in western blot assay.

### *In vivo* SPECT/CT imaging of ^99m^Tc-HMPAO-ENVs

All animal experiments were conducted according to the protocols approved by the Institutional Animal Care and Use Committee at Seoul national university hospital, Seoul, Republic of Korea (Approval number: 13-0265-COA1). 8 week-old male BALB/c mice (Koatech, Seoul, Korea) were bred in a pathogen-free facility at Seoul National University Hospital Biomedical Research Institute. Weight-matched mice were housed in laboratory animal care facility in cages (5 mice/cage) and fed a standard diet. For isolation of ^99m^Tc-HMAPO-ENVs, the exosome exclusive spin column was used as aforementioned. 7.4–14.8 MBq of 29–64 μg ^99m^Tc-HMPAO-ENVs prepared by Raw 264.7 cells (n = 3) and 11.1 MBq of ^99m^Tc-HMPAO (n = 1) were injected into the tail vein. In addition, 4.4 MBq of ^99m^Tc-HMPAO-ENVs prepared from HB1.F3 cells (30–35 μg) were also injected (n = 2). After the administration of ^99m^Tc-HMPAO-ENVs, serial SPECT/CT scans were acquired on a dedicated SPECT/CT scanner (NanoSPECT/CT, Bioscan, Washington, DC). All mice were anesthetized and maintained with 1.5% isoflurane at 1 L/min oxygen flow and placed on the prone position. The four multipinhole γ-detectors (9-pinholes) were used for high contrast collimation. Additional CT scans were acquired after SPECT acquisition for anatomical localization. Acquisition time was adjusted according to the counts to be more than 30,000 counts per projection. For SPECT images, 24 projections were obtained into an 80 × 80 acquisition matrix. The images were reconstructed by a 3-dimensional ordered-subsets expectation maximum (OSEM) algorithm. The radioactivity in the organs was calculated in the SPECT images by volume-of-interest analysis. Counts were corrected for the decay of radioisotope and normalized by percent injected dose per gram (%ID/g).

### *In vivo* biodistribution of ^99m^Tc-HMPAO-ENVs

For biodistribution study, 74 kBq of ^99m^Tc-HMPAO-ENVs prepared by Raw 264.7 cells was injected into the tail vein of the mice (BALB/c, 7–8 weeks old, n = 15). Mice were sacrificed after 0.5, 1, 3, and 5 h. Heart, lung, skeletal muscle, liver, spleen, stomach, intestine, kidneys, brain, bone and blood samples were taken and weighed. The radioactivity of organs was determined by scintillation counting. The results were expressed as %ID/g.

### Preparation of natural exosomes and SPECT/CT images

We extracted natural exosomes from Raw 264.7 cells. Complete DMEM cell medium was harvested after Raw 264.7 cells were incubated for 24 hours with exosome-depleted FBS prepared by ultracentrifugation at 150,000 G for overnight. Cell debris from harvested cell medium was removed by serial centrifugation at 3,000 g for 15 min and 3,000 g for 20 min. Natural exosomes were purified by ultracentrifugation at 150,000 g for 2 hours at 4 °C. The collected exosome pellets were dissolved with PBS and the produced natural exosomes were stored at −70 °C. The radiolabeling procedures were same as those of ENVs. 0.74 MBq of 15 μg ^99m^Tc-HMPAO labeled natural were injected into a BALB/c mouse and acquired SPECT/CT images as ^99m^Tc-HMPAO ENVs SPECT/CT at 3 hr after the injection.

### Ethics statement

All animal experiments were conducted according to the protocols approved by the Institutional Animal Care and Use Committee at Seoul national university hospital, Seoul, Republic of Korea (Approval number: 13-0265-COA1).

## Additional Information

**How to cite this article**: Hwang, D. W. *et al*. Noninvasive imaging of radiolabeled exosome-mimetic nanovesicle using ^99m^Tc-HMPAO. *Sci. Rep*. **5**, 15636; doi: 10.1038/srep15636 (2015).

## Supplementary Material

Supplementary Information

## Figures and Tables

**Figure 1:**
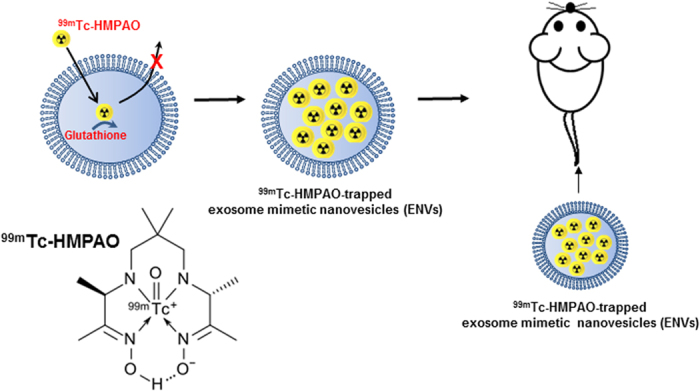
Schematic diagram for radiolabeling method of ^99m^Tc-HMPAO-exosome nanovesicles (ENVs). ^99m^Tc-hexamethylpropyleneamineoxime (HMPAO) was chosen as one of the proper radiotracers for exosome labeling. After highly lipophilic ^99m^Tc-HMPAO enters the ENVs, endogenous glutathione in ENVs begins to convert ^99m^Tc-HMPAO to hydrophilic form, which is trapped inside ENVs.

**Table 1:** Radiochemical purity and protocol time for each isolation method.

Methods	*Procedure time	**Radiochemical purity (%) (n = 3/group)
**Elution over PD-10 column**	30 min	99.6±3.3
**Centrifugation with exosome exclusive spin column**	3 min	93.7±5.4

^*^Procedure time: the time point until ^99m^Tc-HMPAO-ENV was produced after the purification via size exclusion PD-10 column or exosome exclusive spin column.

^**^Radiochemical purity: the measured % values by ITLC using Whatman no. 1 paper and 0.9% w/v NaCl solution as an eluent. Radiochemical purity was determined by following equation: E(%) = (1 − radioactivity of supernatant)/total radioactivity × 100%.
