# Supplementary material for: Noninvasive imaging of radiolabeled exosome-mimetic nanovesicle using 99mTc-HMPAO
Source: Sci Rep. 2015 Oct 26;5:15636. doi: 10.1038/srep15636 (PMC4620485; doi:10.1038/srep15636)
Supplement: Supplementary Information [file srep15636-s1.pdf]

Supplementary Information

Noninvasive imaging of radiolabeled exosome-mimetic nanovesicle using ^{99m}Tc-HMPAO

Do Won Hwang[1,2,†], Hongyoon Choi[1,2,†], Su Chul Jang[3,4], Min Young Yoo[1], Ji Yong Park[1], Na Eun Choi[1], Hyun Jeong Oh[1,2], Seunggyun Ha[1,2], Yun-Sang Lee[1,2], Jae Min Jeong[1], Yong Song Gho[3,*], Dong Soo Lee[1,2,*]

[1]Department of Nuclear Medicine, Seoul National University College of Medicine, Seoul, Korea, [2]Department of Molecular Medicine and Biopharmaceutical Sciences, Graduate School of Convergence Science and Technology, and College of Medicine or College of Pharmacy, Seoul National University, Seoul, Korea, [3]Department of Life Sciences, Pohang University of Science and Technology, Pohang, Gyeongbuk 790-784, Korea, [4]Krefting Research Centre, Department of Internal Medicine and Clinical Nutrition, University of Gothenburg, 405 30 Gothenburg, Sweden

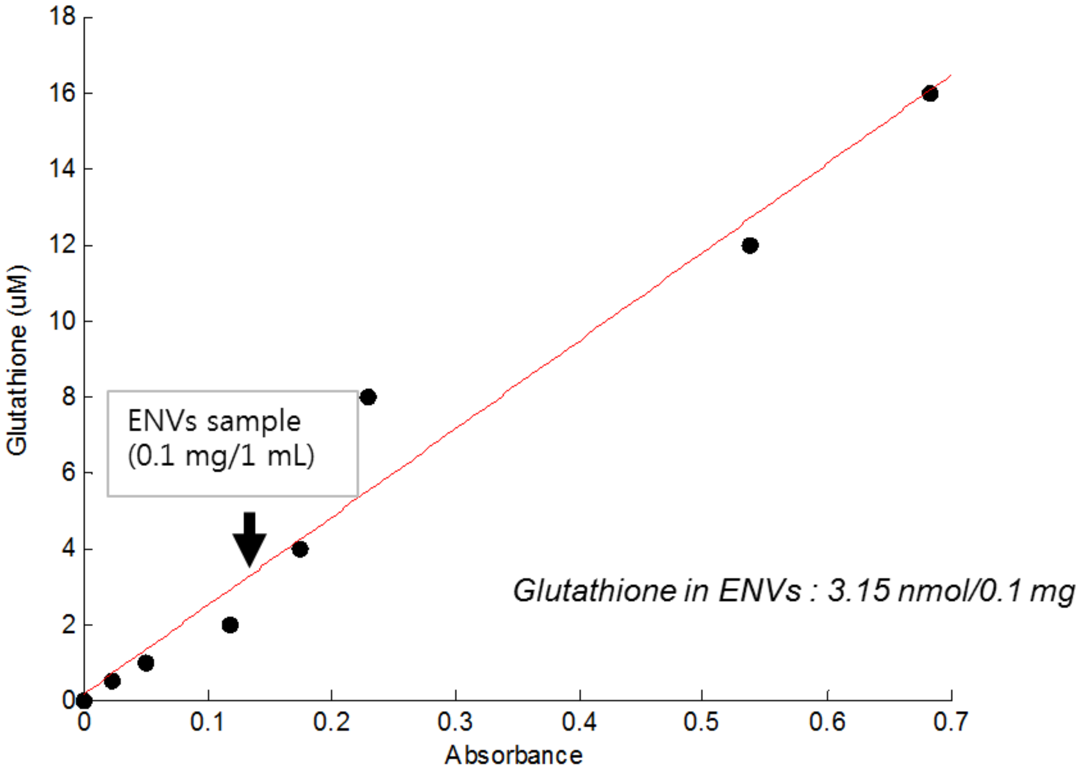

Supplementary Figure 1. Glutathione assay for ENVs. We checked whether ENVs carried endogeneous glutathione. Glutathione assay was carried out using enzymatic reaction. In 0.1 mg of ENV sample, 3.15 ± 0.16 nmol of glutathione was detected. Red-line represents standard curve obtained from known glutathione concentration (n = 4).

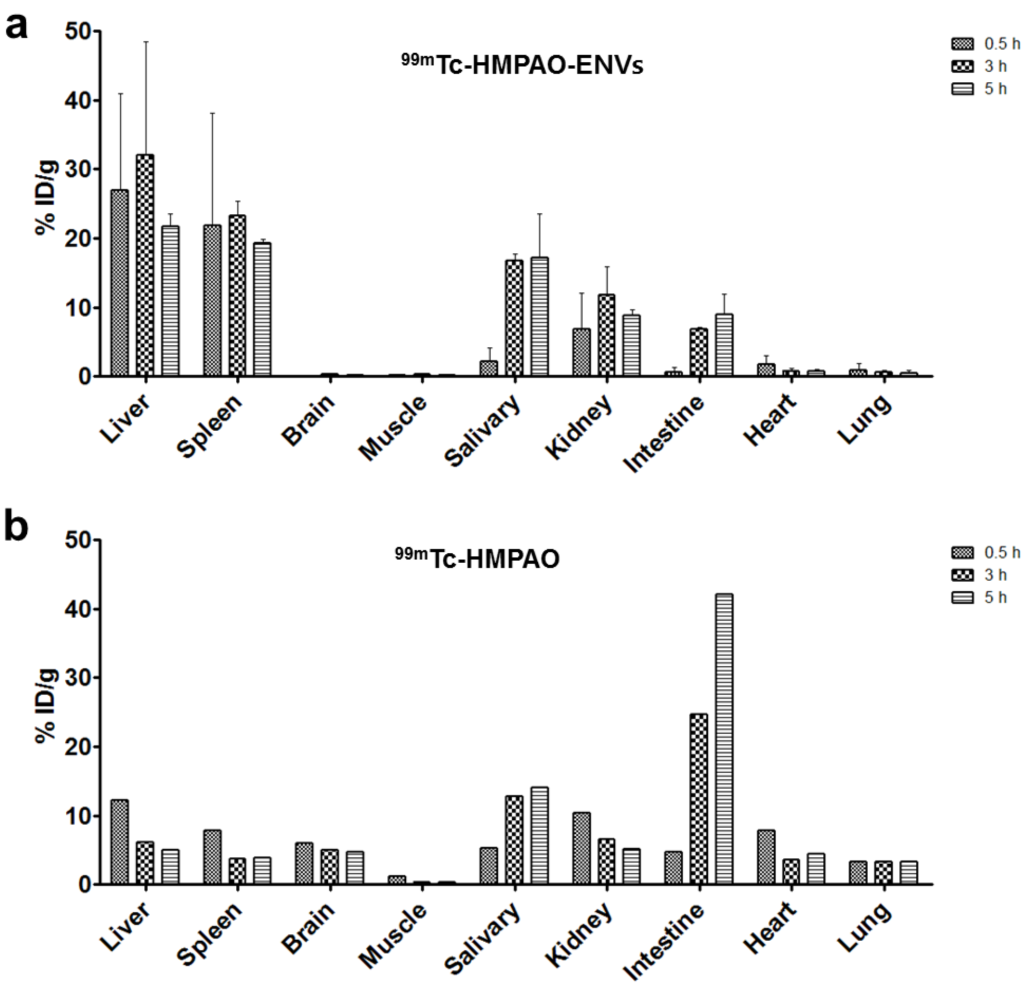

Supplementary Figure 2. Volume-of-interest analysis of SPECT images. The %ID/organ values from the SPECT/CT images were calculated, showing high radioactivity in the liver and spleen until 5 hr. In contrast, the %ID/g values of ^{99m}Tc-HMAPO for the liver and spleen uptake showed maximum uptake at 30 min and then decreased until 5 hr (n = 3).

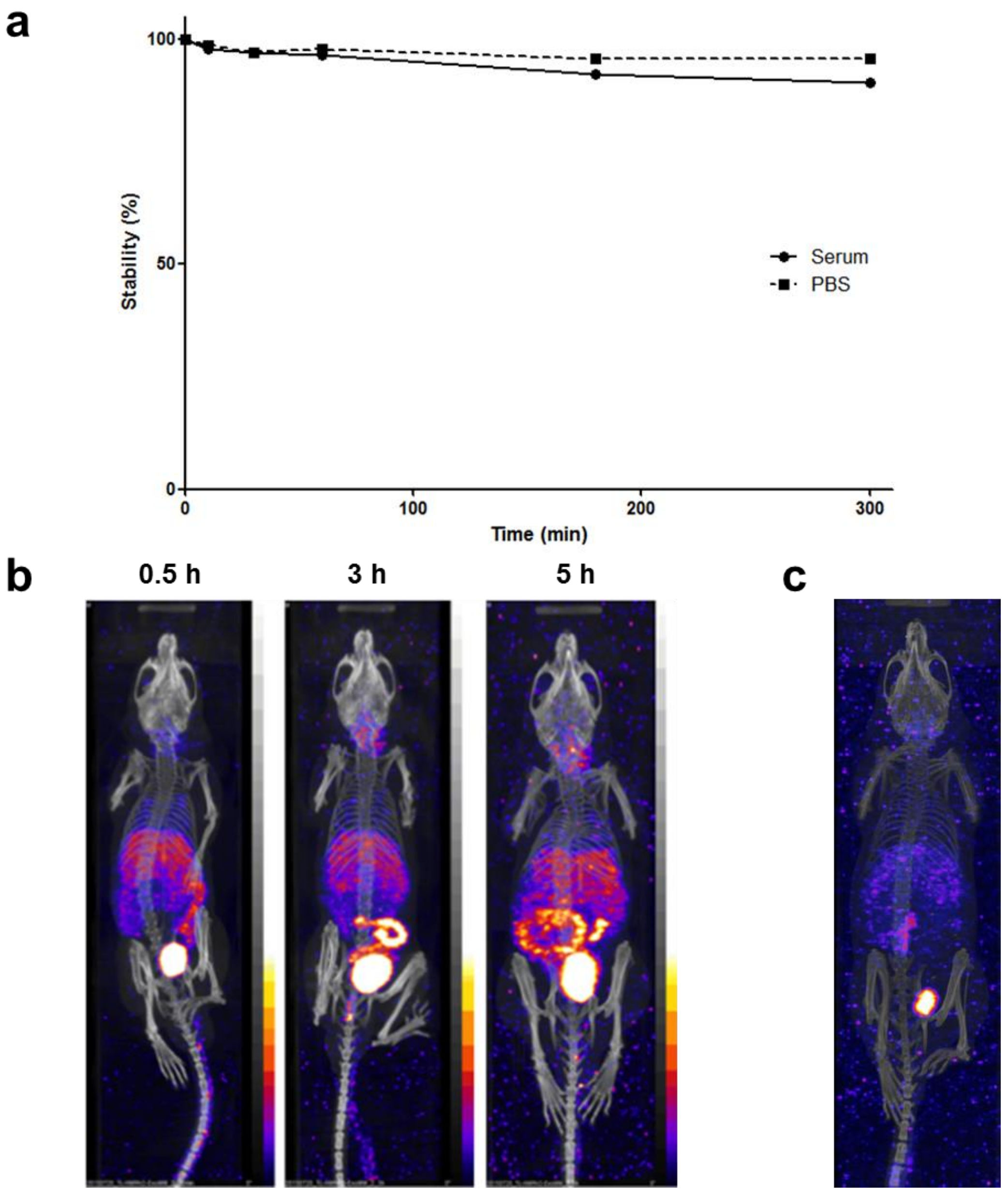

Supplementary Figure 3. ***In vivo*** **SPECT/CT images of ^{99m}Tc-HMPAO-ENVs obtained from human neural stem cells and ^{99m}Tc-HMPAO labeled natural exosomes.** ^{99m}Tc-HMPAO-ENVs were prepared from different cells, HB1.F3 human neural stem cells and SPECT/CT images were acquired in BALB/c mice. (a) Stability of ^{99m}Tc-HMPAO-ENVs was also examined in human serum and PBS. (b) The SPECT/CT images showed similar

distribution pattern with ENVs obtained from Raw 264.7 murine macrophage cells. Intense uptake was found in the liver and spleen within 30 min and radioactivity was gradually found in the salivary glands and intestine. (c) SPECT/CT images of ^{99m}Tc-HMPAO labeled natural exosomes obtained from Raw 264.7 cells at 3 hr revealed similar accumulation patterns with ^{99m}Tc-HMPAO-ENVs.

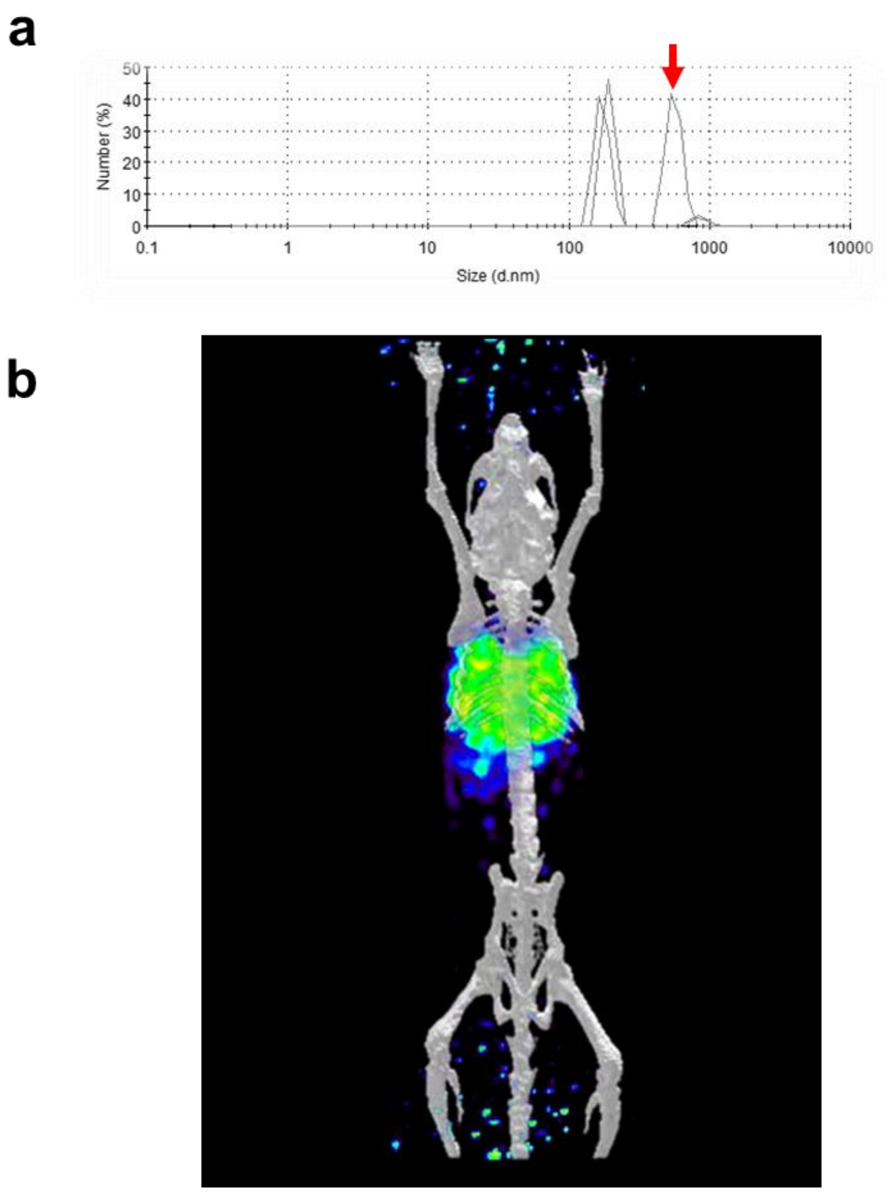

Supplementary Figure 4. Effects of increased size of ENVs in biodistribution. (a) When ^{68}Ga labeling to ENVs via SCN-Bn-NOTA chelator was tried, size of ENVs increased to 530 nm (arrow) probably due to aggregations contrary to the slight increase in ^{99m}Tc-HMPAO labeling. (b) ^{68}Ga-ENVs with increased size resulted in distinct distribution, mainly accumulation in lung (1 hr after injection).
